# Recovery of Added-Value Compounds from Orange and Spinach Processing Residues: Green Extraction of Phenolic Compounds and Evaluation of Antioxidant Activity

**DOI:** 10.3390/antiox10111800

**Published:** 2021-11-11

**Authors:** María Fernanda Montenegro-Landívar, Paulina Tapia-Quirós, Xanel Vecino, Mònica Reig, César Valderrama, Mercè Granados, José Luis Cortina, Javier Saurina

**Affiliations:** 1 Chemical Engineering Department, East Barcelona Engineering School (EEBE), Campus Diagonal-Besòs, Polytechnical University of Catalonia (UPC)-BarcelonaTECH, C/ Eduard Maristany 10-14, 08930 Barcelona, Spain; mafernandy-@hotmail.com (M.F.M.-L.); pautapqui@yahoo.com.mx (P.T.-Q.); xanel.vecino@upc.edu (X.V.); monica.reig@upc.edu (M.R.); cesar.alberto.valderrama@upc.edu (C.V.); jose.luis.cortina@upc.edu (J.L.C.); 2 Barcelona Research Center for Multiscale Science and Engineering, Campus Diagonal-Besòs, 08930 Barcelona, Spain; 3 Chemical Engineering Department, School of Industrial Engineering-CINTECX, Campus As Lagoas-Marcosende, University of Vigo, 36310 Vigo, Spain; 4 Department of Chemical Engineering and Analytical Chemistry, Universitat de Barcelona, Diagonal 645, 08028 Barcelona, Spain; mgranados@ub.edu; 5 CETAQUA, Carretera d’Esplugues, 75, 08940 Cornellà de Llobregat, Spain

**Keywords:** agri-food wastes, mechanical stirring extraction, antioxidant activity, waste to resources, resource recovery

## Abstract

Phenolic compounds recovery by mechanical stirring extraction (MSE) was studied from orange and spinach wastes using water as a solvent. The statistical analysis showed that the highest total polyphenol content (TPC) yield was obtained using 15 min, 70 °C, 1:100 (*w/v*) solid/solvent ratio and pH 4 for orange; and 5 min, 50 °C, 1:50 (*w/v*) solid/solvent ratio and pH 6 for spinach. Under these conditions, the TPC was 1 mg gallic acid equivalent (GAE) g^−1^ fresh weight (fw) and 0.8 mg GAE g^−1^ fw for orange and spinach, respectively. MSE substantially increased the phenolic compounds yields (1-fold for orange and 2-fold for spinach) compared with ultrasound-assisted extraction. Furthermore, the antioxidant activity of orange and spinach extracts was evaluated using DPPH, FRAP and ABTS. The obtained results pointed out that the evaluated orange and spinach residues provided extracts with antioxidant activity (2.27 mg TE g^−1^ and 0.04 mg TE g^−1^, respectively).

## 1. Introduction

Fruits and vegetables are a rich source of phenolic compounds that provide the plant with protection against harmful ultraviolet radiation and pathogens, among other abiotic and biotic stresses [1,2,3,4]. Phenolic compounds are secondary metabolites produced by plants. These act on the plant defense mechanism against, e.g., insects, fungi, drought, and extreme temperatures, among other stress factors [5]. Phenolic compounds, including flavonoids, phenolic acids and others [6], display great antioxidant power (biological and free radical scavenging activity) as one of their principal properties [7], being the reason that the recovery of phenolic compounds has become a key strategy to satisfy the growing demand from the food, cosmetic and pharmaceutical industries [8]. For example, natural antioxidants have been used to replace the synthetic antioxidants additives (e.g., butylated hydroxytoluene (BTH) or butylated hydroxyanisole (BHA)) used in food products [9,10,11], since their toxicity has been proven [12,13].

Another source of phenolic compounds is the agri-food processing industries that generate large amounts of by-products and/or wastes (e.g., seeds and peels of *Citrus* spp., olive mill wastewater, artichoke leaves and stem) [14]. Orange (*Citrus sinensis*) and spinach (*Spinacia oleracea*) crops are among the most abundant in Spain, generating between 50% and 13% of wastes, respectively [15]. It is well established that orange, spinach and their by-products are rich sources of minerals, vitamins and dietary fiber as well as bioactive compounds like polyphenols (specifically flavonoids and phenolic acids) [16], which also provide a high antioxidant activity [8,11]. 

The antioxidant activity of phenolic compounds is linked to their structure; they generally act by preventing the formation of free radicals involved in the autoxidation process, for which they donate electrons or hydrogen atoms or chelating metal cations [14]. Many studies have found that orange and spinach have antioxidant phenolic compounds (e.g., ferulic acid, luteolin, hesperidin) with promising effects in various diseases such as diabetes, cancer, and hypertension, among others [17,18].

The growing interest for the recovery of phenolic compounds from agri-food wastes and their use as ingredients in cosmetic, pharmaceutical and food preparations has led to develop efficient and cost-effective extraction processes. In this regard, mechanical stirring extraction (MSE) uses low temperatures, requires a simple equipment and the process is not expensive [19]. MSE follows the procedure of shaking the sample in contact with a solvent for a certain time and at a certain temperature to preserve the stability of phenolic compounds [20,21]. The advantage of agitation is that facilitates extraction by increasing diffusion and removing concentrated solution from the sample surface to bring new solvent, and thus achieves an elevated extraction performance [22]. Additionally, the solvent nature plays an important role to obtain a high extraction yield. Methanol, acetone, and ethanol are the most used. Despite their effectiveness, from an industrial point of view, cost, toxicity and safety of other solvents should be considered such as water for high-volume extraction [23,24,25]. According to Gómez-Mejía et al. [26], research should focus on how to improve the efficiency of aqueous extraction. Additionally, the development of an efficient, energy-saving, and sustainable processes, can also offer advantages to the food industry in terms of energy consumption, time and profitability. In this way, a cleaner production of phenolic compounds can be achieved and thus to achieve a high demand.

Therefore, in view of the above, the aim of the present work is to optimize the phenolic compounds extraction with water, as a solvent, from orange and spinach wastes by mechanical stirring extraction (MSE) and to compare the selected conditions with ultrasound-assisted extraction (UAE), which is one of the most widely used technique for these purposes [17,27]. The total polyphenolic content (TPC) was determined by Folin–Ciocalteu (FC) and by the high-performance liquid chromatography (HPLC-DAD). Furthermore, the antioxidant activity of several fruit and vegetables residues was evaluated by different tests including 2,2-diphenyl-1-picrylhydrazyl (DPPH), ferric reducing antioxidant power (FRAP) and 2,2′-azino-bis(3-ethylbenzothiazoline-6-sulfonic) acid (ABTS).

## 2. Materials and Methods

### 2.1. Reagents and Solvents

Phenolic compounds used as standards were as follows: 3,4-dihydroxybenzoic acid (>97%), 4-hydroxybenzoic acid (99%), ferulic acid (99%), gallic acid (>97.5), naringenin (>97%), *p*-coumaric acid (>97%), rutin (>94%), syringic acid (>95%), caffeic acid (>98%), and vanillic acid (97%), from Sigma Aldrich (St. Louis, MO, USA); hesperidin (>90%) from Glentham Life Sciences (Corsham, UK); and 6-hydroxy-2,5,7,8-tetramethylchroman-2-carboxylic acid (Trolox, 98% purity) was purchased from Carbosynth (Berkshire, UK).

Acetonitrile (ACN, HPLC grade, >99) was purchased from Fisher Scientific (Loughborough, UK). Ethanol (EtOH, HPLC grade), formic acid (98–100% *w/w*) and hydrochloric acid (32% *w/w*) were obtained by Merck (Darmstradt, Germany). Water was purified with a Milli-Q equipment (Merck Millipore, Burlington, MA, USA).

The chemicals used in antioxidant index tests were as follows: formic acid (98–100% *w*/*w*) and potassium peroxodisulfate (>99%) from Sigma Aldrich (St. Louis, MO, USA), hydrochloric acid (32%, *w/w*), sodium hydroxide (>99%), Fe (III) chloride (>99%), sodium carbonate (>99%) and disodium hydrogen phosphate (>99%) from Merck (Darmstradt, Germany); Folin-Ciocalteu (FC) reagent was a commercial solution ready to use from Panreac; 2,2′-azino-bis(3-ethylbenzothiazoline-6-sulfonic) acid (ABTS, 98%), 2,2-diphenyl-1-picrylhydrazyl (DPPH, 95%) and 2,4,6-tripyridyl-S-triazine (TPTZ, 99%) from Alfa Aesar (Kandel, Germany).

### 2.2. Fruit and Vegetable Samples

Orange (*Citrus sinensis*), kiwi (*Actinidia sinensis*), white and red grape (*Vitis vinifera*), strawberry (*Fragaria vesca*), spinach (*Spinacia oleracea*), carrot (*Daucus carota*), celery (*Apium graveolens*), beet (*Beta vulgaris*), kale (*Brassica oleracea var. sabellica*) and broccoli (*Brassica oleracea var. italica*) were purchased from a local market (Barcelona, Spain). One kg of each one was used in the process of simulating the obtaining of waste from the agri-food industries, specifically in the juice processing. Fruits and vegetables were processed with a domestic juicer. The solid residues obtained (such as orange peel and seeds, and spinach leave waste) were used as representative waste samples and stored in the freezer at −20 °C.

### 2.3. Instruments and Lab Equipment

The phenolic compounds were determined by HPLC-DAD, with an Agilent Series 1200 HPLC chromatography (Agilent Technologies, Palo Alto, CA, USA) with a quaternary pump (G1311A), a degasser (G1322A), an automatic injection system (G1392A) and a diode array detector (G1315B). The Agilent ChemStation software was used for instrument control and data processing.

The antioxidant and antiradical capacities of vegetable and fruit extracts from the set of samples given in Section 2.2 were estimated with a double beam Perkin Elmer UV/Vis/NIR Lambda 19 spectrophotometer. QS quartz glass high performance cuvettes (10 mm optical path) from Hellma Analytics (Jena, Germany) were used.

The extraction of phenolic compounds was carried out using a magnetic stirred furnished with a heating plate (IKA RCT basic, Staufen, Germany). The pH was measured using a pH-meter from Crison (Alella, Barcelona, Spain). On the other hand, the UAE of phenolic compounds was conducted using an ultrasonic bath (Branson 5510, Danbury, CT, USA). The obtained extracts were centrifuged (Rotina 420, Hettich, Tuttlingen, Germany) and the supernatant was filtered through 0.45 µm nylon filters (Whatman, Clifton, NJ, USA).

### 2.4. Extraction of Phenolic Compounds

#### 2.4.1. Mechanical Stirring Extraction (MSE)

The samples were treated using the conditions listed in Table S1. In brief, 1 g of each by-product sample was mixed with the solvent (Milli-Q water) and placed in the stirring plate. The extraction variables were contact time (5, 15 and 30 min), temperature (25, 50, 70 and 90 °C), solid/solvent ratio (1:10, 1:30, 1:50, 1:100 and 1:200 (*w/v*)), and pH (3, unadjusted, and 10). For each assayed condition, experiments were performed in triplicate. After MSE treatment, the resulting extracts were centrifuged for 15 min at 3500 rpm. The supernatant was filtered using a nylon membrane of 0.45 µm. The extracts were stored at 4 °C until the chromatographic and antioxidant analysis.

#### 2.4.2. Ultrasound-Assisted Extraction (UAE)

Extractions were performed the under optimized conditions from previous works [15]. For orange residue, 60:39.9:0.1 ethanol:water:HCl (*v/v/v*) solvent and contact time of 30 min at 25 °C, and for spinach residue 80:19.9:0.1 ethanol:water:HCl (*v/v/v*) solvent for an extraction time of 30 min at 25 °C (frequency of 42 kHz and power of 135 W) were used. Briefly, 1 g of orange and spinach samples was mixed with 20 mL of solvent and sonicated. After that, the mixture was centrifuged for 15 min at 3500 rpm. The supernatant was filtered through 0.45 µm nylon filters. The extracts were stored at 4 °C until the chromatographic and antioxidant analysis. Determinations were carried out in triplicate.

### 2.5. Antioxidant Activity Evaluation

The antioxidant activity of the extracts was determined according to an adaptation of the DPPH, FRAP, ABTS and FC methods described by Alcalde et al. [28].

#### 2.5.1. DPPH

A 0.2 mM DPPH stock solution in 50 mL ethanol was prepared and kept in dark for 2 h. Then, 2 mL of the DPPH solution, 0.8 mL of 0.2 M phosphate buffer (pH 7.4), the necessary volume of standard/sample and Milli-Q water up to 4 mL were mixed and kept in dark for 45 min. The absorbance was recorded at 517 nm using a reagent blank as the reference (the blank absorbance versus water was ca. 1.0 AU). The calibration range was from 0.2 to 10 mg L^−1^ Trolox (R^2^ = 0.984). The DPPH values were expressed as mg Trolox equivalents/g of fresh weight using the standard curve established previously. All samples were analyzed in duplicate.

#### 2.5.2. FRAP

Required volume of the standard/sample was mixed with 300 µL of FRAP reagent consisting of 20 mM L^−1^ FeCl_3_, 10 mM L^−1^ TPTZ (containing 50 mM L^−1^ HCl) and 50 mM L^−1^ formic acid solution in a proportion of 1:2:10 (*v/v/v*) and up to 2.5 mL with Milli-Q water. The absorbance was recorded at 595 nm resulting after 5 min of the reaction, using a blank as the reference. The calibration range was 0.2 to 5 mg L^−1^ Trolox (R^2^ = 0.999). The FRAP values were expressed as mg Trolox equivalents/g of fresh weight using the standard curve established previously. All samples were analyzed in duplicate.

#### 2.5.3. ABTS

ABTS^•+^ reagent was generated with 20 mL of 7 mM ABTS and 350 µL of 140 mM potassium peroxodisulfate. The mixture was kept in the dark for 16 h before use. A daily working solution was prepared with 600 µL of ABTS^•+^ in 24 mL of EtOH. Then, 1.5 mL of ABTS^•+^ was diluted in the required volume standard/sample and measure up to 2.5 mL with Milli-Q water. The absorbance was measured at 734 nm using the reagent blank as the reference after 25 min of the reaction time. The calibration range was 0.2 to 10 mg L^−1^ Trolox (R^2^ = 0.906). The ABTS values were expressed as mg Trolox equivalents g^−1^ of fresh weight using the standard curve established previously. All samples were analyzed in duplicate.

#### 2.5.4. FC Assay

Required volume of standard/sample was mixed with 250 µL of commercial FC reagent. After 8 min, 75 µL of 7.5% (*w/v*) sodium carbonate aqueous solution and Milli-Q water up to 5 mL were added. The reaction was developed for 2 h and the absorbance was recorded at 765 nm in front of the reagent blank as the reference. TPC was expressed as mg gallic acid equivalents per g of fresh weight (mg GAE g^−1^ fw), and the calibration range was from 1 to 20 mg L^−1^ GAE (R^2^ = 0.966). All samples were analyzed in duplicate.

### 2.6. Polyphenolic Content Determination by HPLC-DAD

The extracts of fruits and vegetables indicated in Section 2.2 were analyzed by HPLC with diode array detection (DAD). A Kinetex C18 column (100 mm × 4.6 mm of internal diameter and 2.6 µm particle size) from Phenomenex (Torrance, CA, USA) was used. The mobile phase was composed of 0.1% formic acid in Milli-Q water (solvent A) and Acetonitrile (solvent B). The gradient elution program was as follows: 0 min, 5% B; 30 min, 20% B; 40 min, 45% B; 40.2 min, 5% B; 50 min, 5% B. The flow rate was 1 mL min^−1^ and the injection volume was 5 µL. Chromatograms were recorded at 280, 310 and 370 nm. The total phenolic content (TPC) was estimated from the chromatograms at 280 nm, in the time window between 5 and 36 min, where elution of polyphenols occurs. It is assumed that the peak area is mostly due to polyphenols, and TPC is expressed in terms of gallic acid equivalents (GAE) per g of fresh weight, by calibrating with gallic acid standards in the concentration range 0.5 to 100 mg L^−1^. In addition, the individual quantification of target analytes, including 4-hydroxybenzoic acid, caffeic acid, ferulic acid, hesperidin, and rutin, was carried out using their corresponding standards in the working range 0.5 to 20 mg L^−1^. The occurrence of these compounds in the matrices under study was confirmed elsewhere by LC-MS (see ref. [15]). Here, they were checked by HPLC-UV based on retention time and UV spectral features compared with those of the corresponding standards.

### 2.7. Design of Experiments (DoE)

The optimization of the orange and spinach wastes extraction by MSE was planned. Four independent variables temperature, time, solid/solvent ratio and pH were screened to select the optimal condition used for the extraction and recovery of phenolic compounds. The analysis of variance (ANOVA) was conducted to ascertain the relevance of factors such as temperature, time, solid/solvent ratio and pH of the matrices (orange or spinach). Differences at *p* ≤ 0.05 were considered statistically significant.

Principal component analysis (PCA), using the PLS-Toolbox (Eigenvector Research, Inc., Manson, WA, USA), was applied to a global characterization of selected fruits and vegetables according to the antioxidant indexes. The data matrix consisted of 22 rows corresponding to 11 waste by-products extracted in duplicate and 8 columns of the corresponding variable (FRAP, FC, ABTS, DPPH, hydroxybenzoic acids (HB), hydroxycinnamic acids (HC), flavonoids (F) and global TPC). Data was auto scaled to equalize the contribution of the different variables to the model.

## 3. Results and Discussion

### 3.1. Optimization of Phenolic Compounds Extraction from Orange and Spinach Wastes

In order to improve the phenolic compounds extraction from orange and spinach wastes, the influence of temperature, time, solid/solvent ratio and pH was assessed. Among these factors, temperature and time were simultaneously studied according to our previous experience in the phenolic compounds extraction from fruit matrices, and taking into account the data reported in literature for similar systems [15,17,26].

#### 3.1.1. Effect of Temperature and Contact Time on TPC

To stablish the optimal MSE conditions for phenolic compounds extraction in orange and spinach, temperature (25, 50, 70 and 90 °C), contact time (5, 15 and 30 min), solid/solvent ratio (1:10, 1:30, 1:50, 1:100 and 1:200 (*w/v*)) and pH (3, 10 and without adjust) were varied and their influence on the TPC was studied. The experimental conditions selected were based on a compromise between experimental effort and quality of results. In the case of temperature, preliminary studies suggested that the optimal range was around 50 to 70 °C, although other conditions were checked as well. For the pH, there was clear evidence of compound degradation when increasing pH above 6 or 7, which was more severe for some kind of extracts. Anyway, in basic medium, phenolic groups are deprotonated, producing anionic species that could better dissolve in the aqueous media. For this reason, pH 10 was investigated as well.

In these studies, the overall area at 280 nm was used as an excellent descriptor of the total phenolic content of extracts. It should be remarked that, for these fruit and vegetable waste matrices, the occurrence of potential interfering (absorbing) species without antioxidant capacity was negligible. In contrast, in other matrices such as tea, coffee, chocolate, rich in other absorbing compounds without antioxidant properties, such as caffeine and theobromine, their contribution to the area at 280 nm should be removed to avoid an overestimation of the antioxidant power. As occurs with other antioxidant indexes such as FC, this overall phenolic concentration was expressed in gallic acid equivalents (GAE) because the content of phenolic acids in these types of samples was relevant. Results are summarized in Tables 1 and 3.

Table 1 shows the Series I of the orange residue, where the TPC values increased with increasing temperature and the maximum yield was achieved at 90 °C (1.40 ± 0.10 mg GAE g^−1^ fw). Anyways, compared to 70 °C (1.33 ± 0.09 mg GAE g^−1^ fw), the differences were not statistically significant (*p* > 0.05).

Regarding the effect of the contact time, from 5 to 15 min, TPC increased with time, while no significant differences occurred when comparing 15 and 30 min (*p* > 0.05). Thus, 15 min is the selected contact time. In addition, no correlation on TPC was detected in the interaction between temperature and contact time factors. A similar trend from Series I was also observed by Gómez-Mejía et al. [26], that indicated that at higher temperature and contact time may facilitate higher phenolic compounds recovery. Authors studied factors like temperature (62 and 90 °C) and contact time (10 and 15 min) on the extraction of phenolic compounds from orange peels by magnetic agitation with aqueous ethanol (20:80 *v/v*), obtaining as a result that 90 °C and 15 min increased the rutin amount extracted (4.7 mg g^−1^).

According to data reported in Table 2 (Series I), the study of the effect of the temperature and time on the TPC from spinach residue, shown an increasing trend on the TPC from 25 to 50 °C, while at higher temperatures (70 and 90 °C) a decrease was found, indicating a possible degradation of some phenolic compounds with temperature. Nevertheless, no correlation was detected on the extraction yield in the interaction between temperature and contact time. Thus, the optimum temperature was set at 50 °C; and regarding the contact time at 5 min, was significantly higher (except at 90 °C). At the selected temperature and contact time, the TPC obtained was 0.75 ± 0.04 mg GAE g^−1^ fw.

Jaime et al. [29] also determined that temperature and contact time influenced on the phenolic compounds extraction yield from spinach leaves using water as an extractant, being 50 °C and 24 h, respectively, the values selected. This represents a longer contact time than that of our study. Dzah et al. [30] mentioned that long extraction times at high temperatures increase the oxidation rate of phenol and decreases the yield of TPC in the extracts. Hence, efficient extraction temperatures that maintain the stability of the polyphenols are required. It is worth mentioning that the sensitivity of a sample to polyphenol degradation induced by temperature, depends on the polyphenol type in the extract, and their biochemical and physicochemical characteristics, as well as on the interaction between the sample and the solvent. Therefore, results from orange and spinach matrices showed that yield increased with temperature and time due to higher solvation and mass transfer [30,31]. In general, studies on the influence of extraction conditions reveal the importance of the microenvironment effects of variables such as temperature, time, and solid–solvent ratio [15,32,33].

#### 3.1.2. Effect of Solid/Solvent Ratio on TPC

Once, the optima temperature and time were selected from orange and spinach matrices, the solid/solvent ratio was studied between 1:10 to 1:200 (*w/v*). As can be seen in the Series II, in Table 1, the TPC increased from 1:10 to 1:30 (*w/v*) solid/solvent ratio and then it stabilized. Therefore, statistically the effect of solid/solvent ratio on the extraction of phenolic compounds was not significant (*p* < 0.05), thus, the selected ratio was 1:100 (0.84 ± 0.06 mg GAE g^−1^ fw). Although, lower solid/solvent ratio (e.g., 1:30 (*w/v*)) could be selected as optimal due to the TPC concentration, but if we take into account the amount of extracted phenolic compounds, the 1:100 (*w/v*) ratio is the most favorable, for example by applying membrane technology, where huge volume of phenolic compounds is needed.

These results are in agreement with the findings of Jovanović et al. [6], who verified an increase in the TPC when increasing the volume of solid/solvent ratio from 1:10 to 1:30 with 50% ethanol using maceration as an extraction technique.

On the other hand, the results of spinach residue from Series II (see Table 2) showed an increase on TPC from 1:10 to 1:50 ratios, and then decreased considerably from 1:100 to 1:200. However, the ANOVA of solid/solvent ratio revealed no significant differences in the TPC values (*p* < 0.05), thus, the 1:50 ratio (0.68 ± 0.04 mg GAE g^−1^ fw) was chosen, since it achieved a considerable TPC value (0.68 ± 0.04 mg GAE g^−1^ fw). Some studies have been performed using different ratios of plant material and extraction solvents (solid/solvent ratio). For example, Bokov et al. [34] used a similar solid/solvent ratio to extract flavonoids from spinach leaves, reporting good performance using the 1:40 ratio. For both agri-food matrices, at 1:200 (*w/v*) ratio, the sample was very diluted, and thus the TPC was below the limit of quantification of the HPLC method (0.5 mg L^−1^).

Besides, the characteristics of the solvent in relation to the treated samples, their proportions, their affinities and the extraction conditions are important parameters that should be considered in order to obtain an efficient extraction. Specifically, apart from improving extraction yields, the knowledge of the optimal amount of solvent to use is of economic relevance [30,35].

#### 3.1.3. Effect of pH on TPC

Once temperature, contact time and solid/solvent ratio were established, the effect of pH on TPC was evaluated. For this purpose, acidified or basified solutions were added to adjust the pH to 3 (with HCl), 4 (this is the pH of the orange waste, without adjust) and 10 (with NaOH).

For the orange waste, temperature of 70 °C, contact time of 15 min and solid/solvent ratio of 1:100 were the optima to carried out the Series III. As can be seen in Table 1 (Series III), pH was no significant (*p* > 0.05) from statistical point of view. Therefore, the pH selected was pH 4 (without adjustment) with a TPC of 1.02 ± 0.09 mg GAE g^−1^ fw. Conversely, as can be seen in Table 2 (Series III), the extraction of phenolic compounds from spinach residue reported significant dependence on the pH (*p* < 0.05). Attributing degradations undergoing at very acidic or basic pH. Therefore, pH 6 (without adjustment) was selected, in this case the TPC was 0.75 ± 0.01 mg GAE g^−1^ fw. These results agree with Li et al. [36,37] who found that pH had a significant effect on TPC.

### 3.2. Total Phenolic Content and Antioxidant Activity of Orange and Spinach Wastes

#### The Total Phenolic Content: MSE vs. UAE

**Figure 1:**
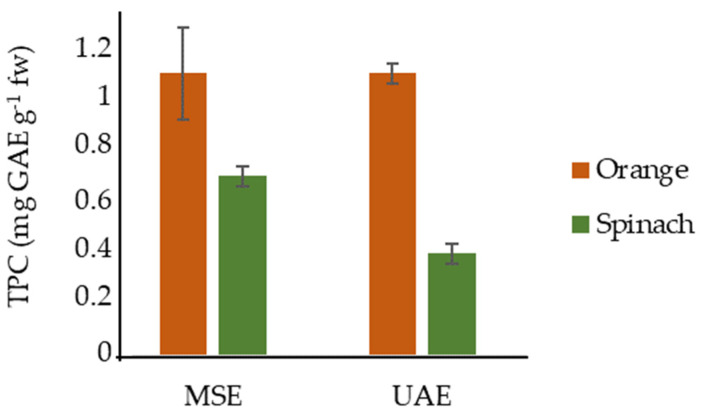
Comparison of TPC yield of MSE and UAE under selected conditions from agri-food residues.

Orange and spinach waste extracts obtained under optima extraction conditions (70 °C, contact time of 15 min, solid/solvent ratio 1:100 and pH 4 without adjustment for orange waste; and 50 °C, 5 min, 1:50 and pH 6 without adjustment, for spinach residue), by DoE approach in terms of TPC, was compared statistically with UAE as can be seen in Figure 1.

Figure 1 shows that the statistical analysis of the data for orange waste do not present significant differences (*p* > 0.05) between MSE and UAE results (1.1 ± 0 mg GAE g^−1^ fw in both case under optimal conditions). Dahmoune et al. [38] also obtained similar results of TPC without statistical differences among MSE and UAE (15.0 and 15.2 mg GAE g^−1^ dw, respectively).

The TPC obtained from spinach residue by MSE (0.75 ± 0.04 mg GAE g^−1^ fw) compared with the TPC of UAE (0.44 ± 0.04 mg GAE g^−1^ fw), was 41% higher using MSE technique (see Figure 1). However, the opposite trend was reported by Altemimi et al. [17] with higher TPC recoveries by UAE than MSE (0.51 and 0.12 mg GAE g^−1^ dw, respectively) from spinach leaves extraction.

In both orange and spinach matrices MSE would be more suitable extraction technique since it is cheaper than UAE. The UAE could be ruled out since unlike the MSE, it applies ultrasonic energy (135 W) and the contact time is longer (30 min), which may cause inconveniences such as polyphenols degradation. In addition, the required equipment and processes with UAE have high costs [14]. Whatever, Gómez-Mejía et al. [26], reported that MSE is fast, sustainable and economic for the extraction of phenolic compounds compared with UAE.

### 3.3. Characterization of Antioxidant Activity of Fruit and Vegetable By-Products Extracts

MSE, under the proposed conditions, seems to be a suitable technique to extract phenolic compounds from orange and spinach wastes compared with UAE (see Figure 1). Other fruits and vegetables by-products were selected to evaluate the antioxidant activity of the extracts by the recommended extraction technique. All of them, including orange, kiwi, strawberry, white and red grape, spinach, carrot, kale, celery, beet and broccoli, were characterized by FRAP, DPPH and ABTS assays expressed as Trolox equivalents (mg TE g^−1^ fw). FC was used to determine the TPC in terms of mg GAE g^−1^ fw.

The natural pH of extract was in the range 3 to 4.5 so that, in some methods, buffer solutions were used to neutralize the excess of acid while providing a proper pH. For a more straightforward procedure focused on routine analysis of large sets of samples, despite the kinetic nature of the reactions absorbances from each index were measured at preselected times leading to steady states. The obtained results of the spectrophotometric assays described in Section 2.5, are reported in Table 3.

A higher value of TE indicates higher antioxidant activity, that is, the samples richest in phenolic compounds present high values for all the indexes and vice versa. In this regarding, it is observed that the most concentrated fruits were orange, white grape and red grape, and for vegetables spinach, kale and beet have the highest antioxidant capacity. In general, Table 3 shows that FRAP generally provides higher values of antioxidant capacity. This may be due to interference issues from non-polyphenolic compounds that may be able to reduce FRAP, but are not as efficient at scavenging radicals. ABTS, as a whole, is the reagent that estimates the lowest antioxidant value, perhaps because it is more stable radical than DPPH.

Moreover, a comparison of data from the four indexes was subjected to a correlation study. For FRAP vs. ABTS reported good correlation coefficient (R^2^ = 0.933), followed by FC vs. ABTS (R^2^ = 0.905), the correlation coefficient indicates that the antioxidant polyphenols that have been involved in one or the other indexes are similar [28]. Therefore, this may indicate the reduction of Fe^3+^ and ABTS^+^ radical (FRAP vs. ABTS) as well as Mo (VI) and Fe (III) (FC vs. ABTS). About the other antioxidant indexes, lower correlations were obtained (see Table S2).

**Figure 2:**
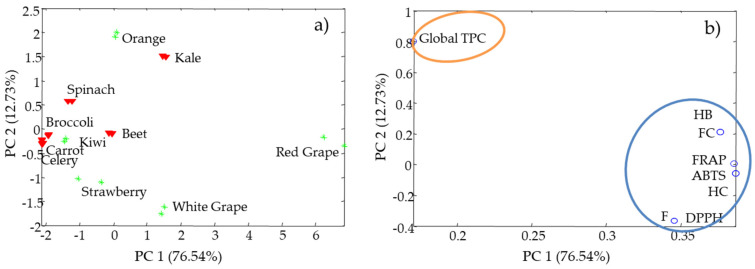
Principal component analysis for the evaluation of the antioxidant features of various fruit and vegetable waste extracts: (**a**) Plot of scores of PC1 vs. PC2 and (**b**) plot of loadings of PC1 vs. PC2. Variable assignation: HB = hydroxybenzoic acids, HC = hydroxycinnamic acids, and F = flavonoids.

In order to summarized and to easily visualize all antioxidant activity results, data was subjected to PCA analysis. The principal components (PCs) are mathematical variables that define efficiently the variation of the data. The first principal component (PC1) explained 76.54% and the second principal component (PC2) explained 12.73 % of the data variance. Relationships between samples and indexes were investigated from the scores and loadings plots (see Figure 2). Scores showed the distribution of extracts with respect to PC1 and PC2 (Figure 2a) and loadings explained the behavior of the variables (Figure 2b). As can be seen in Figure 2a, the samples with low activity (e.g., celery and broccoli) are highly grouped due to the fact that they present few differences between them. On the other hand, samples with higher index values (e.g., red grape, orange and kale) come out to the right and with a lot of dispersion.

Otherwise, Figure 2b provides information on the correlation between the variables. The PC1 and PC2 loadings graphic shows an evident separation between Global TPC and the rest of the variables. Therefore, TPC determines the different behavior between the indexes.

Simultaneous interpretation of the scores and loadings plots suggests that, in Figure 2a, the samples that appear on the right side are the richest in antioxidant compounds. The samples on the left side are poorer in these compounds. Therefore, PC1 explains the antioxidant behavior of the samples. Figure 2b, the samples that are in the upper part show greater radical activity compared to those that are in the lower part.

Regarding to MSE, it seems to be a good technique to extract antioxidant compounds from fruit and vegetables wastes, especially orange and spinach matrices, a very similar index values to that obtained with UAE with ethanol-water mixture by Montenegro-Landívar et al. [15].

### 3.4. Characterization of the Phenolic Composition from Orange and Spinach Wastes

Complementary analyses by HPLC-DAD were performed to identify tentatively various phenolic compounds from orange and spinach waste extracts by MSE under the selected conditions (see in Figure S1). In a previous study by Montenegro et al., the principal molecules in these matrices were identified by LC-MS [15]. In this paper, based on those results, compounds were identified tentatively by HPLC-DAD, from the coincidence of retention times and the UV spectra of suspected compounds, with those of the corresponding standards (see Figure S1 in the supplementary material). The identified phenolic compounds can be allocated into three groups: hydroxybenzoic acids, hydroxycinnamic acids and flavonoids as can be seen in Table 4.

Therefore, as derived from HPLC analysis evaluated in orange and spinach waste extracts (see Figure S1), orange waste could be a rich source of 4-hydroxibenzoic acid and hesperidin. Similar results were obtained by Senit et al. [39]. They reported phenolic acids and flavonoids present in orange peel waste with a remarkable antioxidant activity. On the other hand, spinach residue could be considered a suitable source of caffeic acid, ferulic acid and rutin (see Table 4) under the selected extraction conditions evaluated in this study. Bokov et al. [34] and Vázquez et al. [11] also detected that ferulic acid and caffeic acid were present in spinach extract. According to Montenegro-Landívar et al. [15], orange and spinach wastes are good sources of phenolic compounds that could be recovered for the application in the cosmetic, pharmaceutical and food industries. In this regard, the green nature of the extraction method, without using any harmful solvent, is a key aspect compatible with the production of raw materials for food supplements, nutraceuticals and drugs. Some representative examples of potential applications proposed by other authors were as follows. Papillo et al. [40] suggested that polyphenol extracts from cocoa hulls, can be microencapsulate in order to have heat-stable functional ingredients for bakery products. They used water as solvent and magnetic stirring extraction technique. Additionally, dietary fibers with polyphenols extracted from mango peels were used as functional ingredients in processed foods, due to their potential health benefits (e.g., regulation of blood glucose level, anticarcinogenic effects, antioxidant property) [41].

## 4. Conclusions

Polyphenol extraction from fruit and vegetable wastes was performed using mechanical stirring as a cost-effective technique where water is used as a solvent. Thermo-mechanical treatments of orange and spinach residues, used as model matrices, were applied to evaluate the effect of temperature, time, solid/solvent ratio and pH. Comparing MSE with UAE, the performance was similar for the orange waste; however, for the spinach residue ca. 2-fold improvement was obtained. Therefore, MSE can be postulated to be an efficient technique for the recovery of phenolic compounds from agri-food residues. The MSE optimal conditions for orange wastes were temperature of 70 °C, solid/solvent ratio 1:100 (*w/v*) and pH 4 (without adjustment) in 15 min of contact time, while for spinach residues were temperature of 50 °C, solid/solvent ratio 1:50 (*w/v*) and pH 6 (without adjustment) for 5 min. Using the proposed extraction process, under the optimal conditions, each gram of orange and spinach wastes allow obtaining approximately 1 mg of 4-hydroxybenzoic acid and 5 mg of hesperidin per gram of orange waste; and 0.1 mg of rutin per gram of spinach residue. Additionally, the orange and spinach residues presented high antioxidant activity (0.51 ± 0.02 mg GAE/g fw and 0.47 ± 0.03 mg GAE/g fw, respectively) in comparison with carrot, celery, kiwi, strawberry and broccoli, and low antioxidant activity than kale, white and red grape. Some advantages of the proposed method deal with the use of a cheap and green extraction procedure for the recovery of polyphenols, combining water as the solvent with the mechanical stirring. Products obtained in this way will be fully compatible with applications to functional foods, animal feed, dietary supplements or cosmetics thanks to their polyphenolic content and antioxidant activity.

## Figures and Tables

**Table 1:** Assessment of the influence of the experimental factors on the TPC recovery from orange residues.

Series I				
Temperature (°C)	Time (min)	Solid/solvent ratio (*w/v*)	pH	TPC (mg GAE g^−1^ fw)
25	5	1:20	4	0.51 ± 0.08 ^aA^
	15			0.76 ± 0.05 ^bA^
	30			0.91 ± 0.02 ^bA^
50	5			0.61 ± 0.04 ^aAB^
	15			1.00 ± 0.07 ^bAB^
	30			1.03 ± 0.04 ^bAB^
70	5			0.65 ± 0.07 ^aBC^
	15			1.10 ± 0.20 ^bBC^
	30			1.33 ± 0.09 ^bBC^
90	5			0.94 ± 0.07 ^aC^
	15			1.40 ± 0.10 ^bC^
	30			1.30 ± 0.10 ^bC^
**Series II**				
Temperature (°C)	Time (min)	Solid/solvent ratio (*w/v*)	pH	TPC (mg GAE g^−1^ fw)
70	15	1:10	4	0.68 ± 0.01 ^a^
		1:30		0.83 ± 0.02 ^a^
		1:50		0.81 ± 0.04 ^a^
		1:100		0.84 ± 0.06 ^a^
		1:200		NQ
**Series III**				
Temperature (°C)	Time (min)	Solid/solvent ratio (*w/v*)	pH	TPC (mg GAE g^−1^ fw)
70	15	1:100	3	0.94 ± 0.02 ^a^
			4	1.02 ± 0.09 ^a^
			10	0.98 ± 0.04 ^a^

Mean values (*n* = 3) followed by same lowercase letter within the same extraction parameter and capital letter in each column showed no statistically significant difference (*p* > 0.05). NQ below the quantification limit.

**Table 2:** Assessment of the influence of the experimental factors on the TPC recovery from the spinach wastes.

Series I				
Temperature (°C)	Time (min)	Solid/solvent ratio (*w/v*)	pH	TPC (mg GAE g^−1^ fw)
25	5	1:20	6	0.65 ± 0.02 ^aAB^
	15			0.58 ± 0.01 ^abAB^
	30			0.47 ± 0.02 ^bAB^
50	5			0.75 ± 0.04 ^aA^
	15			0.58 ± 0.02 ^abA^
	30			0.51 ± 0.01 ^bA^
70	5			0.51 ± 0.03 ^aB^
	15			0.48 ± 0.02 ^abB^
	30			0.46 ± 0.00 ^bB^
90	5			0.40 ± 0.01 ^aC^
	15			0.43 ± 0.01 ^abC^
	30			0.30 ± 0.02 ^bC^
**Series II**				
Temperature (°C)	Time (min)	Solid/solvent ratio (*w/v*)	pH	TPC (mg GAE g^−1^ fw)
50	5	1:10	6	0.59 ± 0.09 ^a^
		1:30		0.67 ± 0.07 ^a^
		1:50		0.68 ± 0.04 ^a^
		1:100		0.52 ± 0.04 ^a^
		1:200		NQ
**Series III**				
Temperature (°C)	Time (min)	Solid/solvent ratio (*w/v*)	pH	TPC (mg GAE g^−1^ fw)
50	5	1:50	3	0.19 ± 0.01 ^a^
			6	0.75 ± 0.01 ^b^
			10	0.48 ± 0.01 ^c^

Values followed by same lowercase letter within the same extraction parameter and capital letter in each column denote nonsignificant difference (*p* < 0.05). NQ below the quantification limit.

**Table 3:** Comparison of TPC and antioxidant activity of different fruit and vegetable extracts. Data are expressed as mean ± standard derivation (*n* = 2).

Waste Extracts	DPPH (mg TE g^−1^ fw)	FRAP (mg TE g^−1^ fw)	ABTS (mg TE g^−1^ fw)	FC (mg GAE g^−1^ fw)
Orange	1.31 ± 0.10	2.27 ± 0.25	0.36 ± 0.04	0.51 ± 0.02
Kiwi	0.58 ± 0.02	0.52 ± 0.02	0.28 ± 0.01	0.36 ± 0.04
Strawberry	2.02 ± 0.02	0.78 ± 0.01	0.40 ± 0.02	0.38 ± 0.05
White grape	3.06 ± 0.05	1.97 ± 0.14	1.47 ± 0.07	0.63 ± 0.11
Red grape	3.96 ± 0.16	8.18 ± 0.28	3.37 ± 0.35	2.24 ± 0.02
Spinach	0.70 ± 0.03	0.04 ± 0.00	0.07 ± 0.04	0.47 ± 0.03
Carrot	0.38 ± 0.01	0.09 ± 0.00	0.05 ± 0.00	0.08 ± 0.01
Kale	0.85 ± 0.06	1.57 ± 0.12	1.47 ± 0.03	1.63 ± 0.03
Celery	0.39 ± 0.02	0.04 ± 0.00	0.04 ± 0.00	0.07 ± 0.00
Beet	0.66 ± 0.01	2.28 ± 0.08	1.32 ± 0.06	0.52 ± 0.01
Broccoli	0.48 ± 0.00	0.07 ± 0.01	0.01 ± 0.00	0.20 ± 0.02

GAE (gallic acid equivalents), fw (fresh weight), TE (Trolox equivalents).

**Table 4:** Identified phenolic compounds in the extracts from orange and spinach matrices, their respective family, structure, and concentration under optima conditions.

Polyphenol Family	Structure	Residues	Concentration (mg g^−1^)
Hydroxybenzoic acids	4-hydroxybenzoic acid 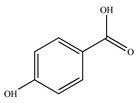	Orange	0.71 ± 0.03
Hydroxycinnamic acids	Caffeic acid 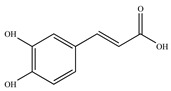	Spinach	0.04 ± 0.01
	Ferulic acid 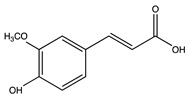	Spinach	0.04 ± 0.01
Flavonoids	Hesperidin 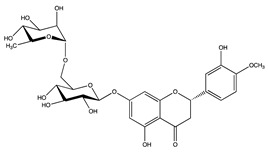	Orange	4.86 ± 0.09
	Rutin 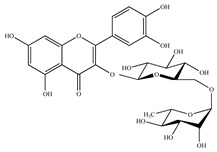	Spinach	0.08 ± 0.01

## Data Availability

Data is contained within the article.

## Supplementary Materials

The following are available online at https://www.mdpi.com/article/10.3390/antiox10111800/s1, Figure S1: Chromatograms of a set of standards at 20 mg L^−1^ each (black lines), an orange extract (brown line), and a spinach extract (green line) recorded at 280 nm to be used for identification purposes. Table S1: Performed variables for the optimization of phenolic compounds extraction, Table S2: Correlation studies among FRAP, DPPH, ABTS and FC.
