# Supplementary material for: Recovery of Added-Value Compounds from Orange and Spinach Processing Residues: Green Extraction of Phenolic Compounds and Evaluation of Antioxidant Activity
Source: Antioxidants (Basel). 2021 Nov 11;10(11):1800. doi: 10.3390/antiox10111800 (PMC8614849; doi:10.3390/antiox10111800)
Supplement: Supplementary file 1 [file antioxidants-10-01800-s001.zip › antioxidants-1441599-supplementary.pdf]

Supplementary material of

Recovery of added-value compounds from orange and spinach processing residues: green extraction of phenolic compounds and evaluation of antioxidant activity

María Fernanda Montenegro-Landívar[1,2], Paulina Tapia-Quirós[1,2], Xanel Vecino[1,2,3]*, Mònica Reig[1,2], César Valderrama[1,2], Mercè Granados[4], José Luis Cortina[1,2,5], Javier Saurina[4]

[1]Chemical Engineering Department, Campus Diagonal-Besòs, Escola d'Enginyeria de Barcelona Est (EEBE), Universitat Politècnica de Catalunya (UPC)-BarcelonaTECH, C/ Eduard Maristany 10-14, 08930 Barcelona, Spain.

[2]Barcelona Research Center for Multiscale Science and Engineering, Campus Diagonal-Besòs, 08930 Barcelona, Spain.

[3]Chemical Engineering Department, Campus As Lagoas-Marcosende, School of Industrial Engineering-CINTECX, University of Vigo, 36310 Vigo, Spain.

[4]Department of Chemical Engineering and Analytical Chemistry, Universitat de Barcelona, Diagonal 645, 08028 Barcelona, Spain.

[5]CETAQUA, Carretera d'Esplugues, 75, 08940 Cornellà de Llobregat, Spain.

**Corresponding author:* xavi.saurina@ub.edu

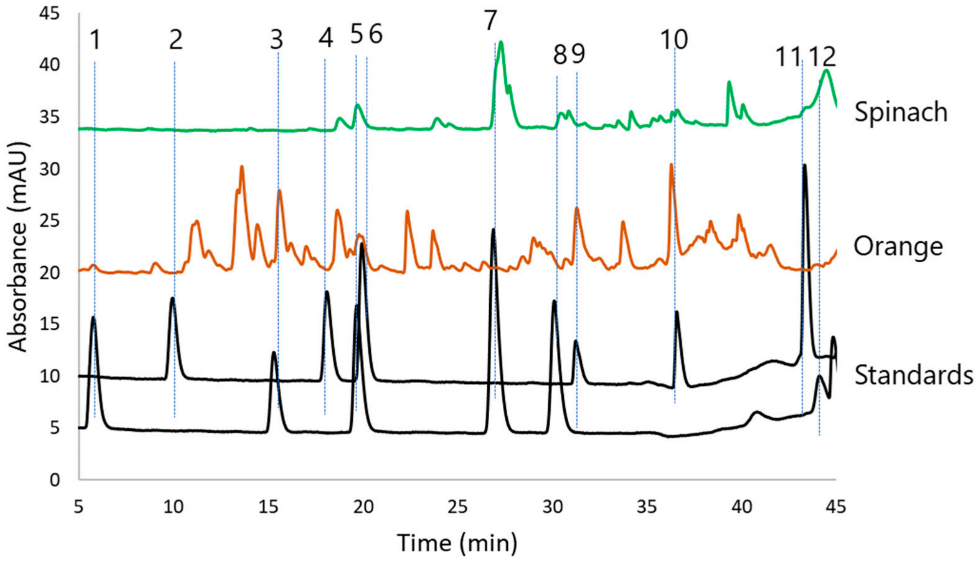

Figure S1: Chromatograms of a set of standards at 20 mg L^{-1} each (black lines), an orange extract (brown line), and a spinach extract (green line) recorded at 280 nm to be used for identification purposes. Selected extraction conditions for orange waste: MSE, temperature 70ºC, solid/solvent ratio 1:100 (w:v) and pH 4, time 15 min. Selected extraction conditions for spinach waste: MSE, temperature 50ºC, solid/solvent ratio 1:50 (w:v) and pH 6, time 5 min). Peak assignment: 1 = Gallic acid, 2 =3,4-Dihydroxybenzoic acid, 3 = 4-Hydroxybenzoic acid, 4 = Syringic acid, 5 = Vanillic acid, 6 = Caffeic acid, 7 = *p*-Coumaric acid, 8 = Ferulic acid, 9 = Rutin, 10 = Hesperidin, 11 = Naringenin, 12 = Kaempferol.

Table S1. Performed variables for the optimization of phenolic compounds extraction.

Assay	R-value
FRAP vs DPPH	0.807
FRAP vs ABTS	0.933
DPPH vs ABTS	0.798
FC vs FRAP	0.845
FC vs DPPH	0.660
FC vs ABTS	0.905

Table S2. Correlation studies among FRAP, DPPH, ABTS and FC.

Experimental variables	Levels	Optima conditions orange waste	Optima conditions spinach residue
Temperature (ºC)	25 / 50 / 70 / 90	70	50
Contact time (min)	5 / 15 / 30	15	5
Solid/solvent ratio *(w:v)*	1:10 / 1:30 / 1:50 / 1:100 / 1:200	1:100	1:50
pH	3 / unadjusted / 10	unadjusted	unadjusted
